# An estimation of the number of children requiring pediatric palliative care in Italy

**DOI:** 10.1186/s13052-020-00952-y

**Published:** 2021-01-07

**Authors:** Franca Benini, Mariadonata Bellentani, Laura Reali, Pierina Lazzarin, Lucia De Zen, Federico Pellegatta, Pierangelo Lora Aprile, Gianlorenzo Scaccabarozzi

**Affiliations:** 1 Pediatric Pain and Palliative Care Service, Department of Women’s and Children’s Health, University Hospital Padua, Via Ospedale 59, Padua, Italy; 2 General Direction of Health Planning of the Italian Ministry of Health, Rome, Italy; 3 ASL RM/E, Rome, Italy; 4 Pediatric Palliative Care and Pain Service, Institute for Maternal and Child Health Burlo Garofolo, Trieste, Italy; 5 Pediatric Hospice “Casa Sollievo Bimbi”, VIDAS Association, Milan, Italy; 6 Italian Society of General Practitioner and Primary Care, Florence, Italy; 7 Department of Fragility, Local Network of Palliative care, ASST Lecco, Merate, Italy

**Keywords:** Pediatric palliative care, Need, Epidemiology

## Abstract

**Background:**

Pediatric palliative care (PPC) addresses the physical and psychological needs of children suffering from life-limiting diseases. To define prevention and educational plans and to properly allocate resources, a precise estimation of the PPC burden is required.

**Objectives:**

To estimate the current number of children requiring PPC in Italy, useful to assist policy-makers and healthcare bodies in the organization and allocation of PPC resources.

**Methods:**

Literature data, The Global Atlas of Palliative Care at the End of Life and Italian national databases have been consulted.

**Results:**

According to our estimation, at present, a total of 20,540–32,864 children in Italy require PPC (34–54 children/100,000 inhabitants) of whom 18 children/100,000 inhabitants require specialized PPC.

**Conclusions:**

The present work is a fundamental tool to be used by the institutions, the local networks of PPC and the health programmers when formulating organizational models and care plans consistent with the actual need for PPC.

**Supplementary Information:**

The online version contains supplementary material available at 10.1186/s13052-020-00952-y.

## Introduction

Science and technology have led to an increase in survival of many children suffering from life-limiting diseases (LLDs). On the other hand, these children live with incurable diseases throughout their lives. Consequently, a new category of pediatric patients with different needs emerged. Pediatric palliative care (PPC) is aimed to address the physical and psychological needs of these children and their families and to provide a major improvement in their quality of life [1, 2] with the definition of new approaches for this peculiar setting [3, 4]. Furthermore, PPC largely differs from adult palliative care (PC), due to the broad spectrum of diseases – many of which are typical in childhood – and the complexity of biological, physical, emotional and spiritual needs of those who are pediatric aged [4].

At present, it is estimated that more than 20 million children are eligible for PPC worldwide, and this figure is likely to increase over the next decade. However, different critical issues to attempt to define PPC needs still exist. Indeed, the lack of homogeneity in the few available data, where the diverse methods for data collection (prevalence or incidence) and the heterogeneity in the eligibility criteria adopted (general or specialized PPC) [4] limit the possibility to compare different numbers.

Consequently, to define prevention and educational plans and to properly allocate PPC resources, a precise estimation of the PPC burden remains an urgent requirement.

A prevalence survey performed in Italy in 2009, with very restrictive inclusion criteria, showed that at least 12,000 children were eligible for PPC (10 minors out of 10,000) [5].

Today, in Italy, the number of children who need PPC is estimated to be more than twice compared to previous estimates, similar to what has happened in other European countries (e.g. UK). However, this estimate remains uncertain and limits, at least in part, the policy-makers in the organization of this fundamental service.

In this paper, we estimate the current number of children requiring PPC in Italy (referred to 100,000 inhabitants), also in comparison to the Italian adult population, with the aim to assist policy-makers and healthcare bodies in the organization and allocation of dedicated resources for PPC.

## Methods

For the present work, we evaluated: (i) the prevalence of needs for PPC and specialized PPC (i.e., PPC in a dedicated setting by a multidisciplinary team of experts in PPC [4]); and (ii) incidence of the need for PPC, using the event of death as indicator and reporting data to a total of 100,000 inhabitants (including both the children and adult populations) and per 10,000 children.

An evaluation of literature (PubMed and Cochrane databases) on PPC and adult PC was conducted, without specific limitations in the search. The search was last updated in August 2020. The Authors’ own personal collections of literature were also browsed.

The Global Atlas of Palliative Care at the End of Life published by The World Health Organization (WHO) and the Worldwide Palliative Care Alliance (WPCA) has also been consulted, even if this document is based only on the need of PC at the end of life (WPCA-WHO Global Atlas of Palliative care at the end of life) [6].

Last, we browsed Italian national databases (Istituto Nazionale di Statistica [ISTAT]) to integrate retrieved data with demographic information for the Italian population.

### Statistical analysis

Descriptive statistics were used for the present study.

## Results

### Estimation of PPC needs, prevalence and incidence

Considering literature data about PC, the prevalence of pediatric and adult patients with PC needs, the proportion of patients who required specialist PC and the incidence of death events in these populations are reported in Table S1 (see supplementary material).

### PPC needs in the Italian scenario

ISTAT data estimate the adult Italian population (aged over 18 years) as 50,089,447 people out of a total of 60,359,546. The death events in Italy were 633,133 in 2018, of which 630,605 deaths were adults.

Considering the Italian pediatric population (0–18 years), this estimate is equal to 10,270,099 children and death events among this population were 2528 in 2018 (almost 50% were aged < 1 year).

The actual prevalence of the pediatric population in Italy with PPC needs, the proportion of patients who required specialist PC and the incidence of death events are reported in Table 1, along with data regarding the adult PC needs.

**Table 1:** Comparison of PC needs in adults and children in Italy

	Pediatric Population	Adult Population
Prevalence of PC needs referred to 100,000 inhabitants of any age	34–54 children per 100,000 inhabitants of any age	830–1100 adults per 100,000 inhabitants of any age
PC prevalence needs referred to 10,000 children or adults	20–32 children per 10,000 children (0–18 years)	100–140 adults per 10,000 adults (> 18 years)
Prevalence of specialized PC needs referred to 100,000 inhabitants of any age	18 children per 100,000 inhabitants of any age Of these: 3 per 100,000 inhabitants are oncological patients 15 per 100,000 inhabitants are non-oncological patients	830–1100 adults per 100,000 inhabitants of any age
Death events in patients with PC needs/year*	*n* = 1706 Children (67.4% of the total*)	*n* = 454,035–504,484 Adults

Total Italian population: 60,359,546 inhabitants (10,270,099 aged 0–18 years, 50,089,447 aged > 18 years [7])

*Total death events in Italy in 2019: 633,133, of which 2528 in the pediatric population (50% of those in patients aged < 1 year); sources: [8])

## Discussion

PPC largely differ from palliative care delivered in the adult setting, due to the heterogeneity of patients and disease, different criteria for the eligibility to PPC, and the diverse needs of pediatric age [4, 9]. Moreover, although the number of adult patients requiring palliative care are undoubtedly more than the number of children in PPC, the journey of a patient within the PPC setting can last for several years. Therefore, dedicated planning and proper allocation of resources is paramount to ensure the best possible management of children in PPC, given also the expected increase of the number of children requiring this medical service in the incoming years [4, 7]. The estimation of the PPC burden represents a necessary step for the allocation of resources by the National Healthcare system. To our knowledge, such estimation is not immediately available for the Italian scenario.

According to our estimation, and taking into account the heterogeneity of data, at present, 34–54 children/100,000 inhabitants of any age in Italy require PPC, accounting for a total of 20,540–32,864 children. Furthermore, 18 children/100,000 inhabitants require specialized PPC, i.e. the delivery of continuous PPC in a dedicated and specialized setting by a multidisciplinary team of experts. These estimations require healthcare regulatory bodies to strengthen healthcare systems through the availability of essential treatments, staff training, public education and community support. Indeed, in this perspective, without improving the integration of PC into healthcare systems, both the inability to alleviate the suffering of patients and the pressure on healthcare systems will increase. In addition, the comparison with the PC demand in adult patients provides a further indication to promote a balanced division of resources between these two settings, which have different specific needs.

## Conclusion

The present paper should be considered by the national and regional institutions, the local networks of PPC and the health programmers of the health institutions when formulating organizational models and care plans consistent with the actual need for PPC.

## Supplementary Information

**Additional file 1: Table S1.** Comparison of PC needs in children and adults.

## Data Availability

The datasets used and/or analyzed during the current study are available from the corresponding author on reasonable request.

## Abbreviations

ISTAT: Istituto Nazionale di Statistica
LLD: life-limiting disease
PC: palliative care
PPC: pediatric palliative care
WHO: World Health Organization
WPCA: Worldwide Palliative Care Alliance
