# Supplementary material for: An estimation of the number of children requiring pediatric palliative care in Italy
Source: Ital J Pediatr. 2021 Jan 7;47:4. doi: 10.1186/s13052-020-00952-y (PMC7791880; doi:10.1186/s13052-020-00952-y)
Supplement: Supplementary file 1 — Additional file 1: Table S1. Comparison of PC needs in children and adults. [file 13052_2020_952_MOESM1_ESM.docx]

**SUPPLEMENTARY MATERIAL**

**The need for pediatric palliative care: an estimation of the number of children requiring pediatric palliative care in Italy**

**Running Title: The need for pediatric palliative care in Italy**

*Franca Benini^*^,* *Mariadonata Bellentan^2^*, *Laura Reali, Pierina Lazzarin*, *Lucia De Zen, Federico Pellegatta, Pierangelo Lora Aprile, Gianlorenzo Scaccabarozzi*

***Corresponding Author**

Franca Benini, Pediatric Pain and Palliative Care Service, Department of Women’s and Children’s Health, University Hospital Padua, Via Ospedale 59, Padua, Italy

**Table S1. Comparison of PC needs in children and adults.**

|  | **Pediatric Population** | **Adult Population** |
| --- | --- | --- |
| PC needs prevalence | 0.2–0.3%  (*Connor et al.; 2017 doi:10.1016/j.jpainsymman.2016.08.020)* | 1−1.4%  *(Gómez-Batiste et al.; 2017 doi:10.1016/j.jpainsymman.2016.10.361)* |
| Specialized PC needs prevalence | 40%  (*Fraser et al.; 2012 doi:10.1542/peds.2011-2846)* | 35%  *(Scaccabarozzi et al.; 2018 doi:10.1089/jpm.2017.0404)* |
| Death events incidence among population with PC needs | 20 death events/year per 100,000 children (ages 0−15 years)  *(WPCA-WHO Global Atlas of Palliative care at the end of life)* | 560 death events/year per 100,000 adults  *(WPCA-WHO Global Atlas of Palliative care at the end of life)* |
|  |  | 69−84% of adult death events/year. (*Murtagh et al.; 2014*  *doi:10.1177/0269216313489367)*  *(Gómez-Batiste et al.; 2012*  *doi:10.1016/j.jpainsymman.2011.05.006)* |
|  |  | 72−80% of total death events  *(Peruselli et al.; 2019*  *DOI 10.1726/3133.31154)* |
